# Silver Nanoparticles Decorated with Curcumin Enhance the Efficacy of Metformin in Diabetic Rats via Suppression of Hepatotoxicity

**DOI:** 10.3390/toxics11100867

**Published:** 2023-10-18

**Authors:** Iftekhar Hassan, Jameel Al-Tamimi, Hossam Ebaid, Mohamed A. Habila, Ibrahim M. Alhazza, Ahmed M. Rady

**Affiliations:** 1 Department of Zoology, College of Science, King Saud University, Riyadh 11451, Saudi Arabia; jhattamimi@gmail.com (J.A.-T.); hossamebaid1969@gmail.com (H.E.); ihazza@ksu.edu.sa (I.M.A.); rady_gad1983@yahoo.com (A.M.R.); 2 Department of Chemistry, College of Science, King Saud University, Riyadh 11451, Saudi Arabia; mhabila@ksu.edu.sa

**Keywords:** silver nanoparticles, curcumin, metformin, diabetes mellitus, hepatotoxicity, oxidative stress

## Abstract

Hepatotoxicity is one of the significant side effects of chronic diabetes mellitus (DM) besides nephrotoxicity and pancreatitis. The management of this disease is much dependent on the restoration of the liver to its maximum functionality, as it is the central metabolic organ that gets severely affected during chronic diabetes. The present study investigates if the silver nanoparticles decorated with curcumin (AgNP-Cur) can enhance the efficacy of metformin (a conventional antidiabetic drug) by countering the drug-induced hepatoxicity. Swiss albino rats were categorized into six treatment groups (*n* = 6): control (group I without any treatment), the remaining five groups (group II, IV, V, VI) were DM-induced by streptozocin. Group II was untreated diabetic positive control, whereas groups III was administered with AgNP-cur (5 mg/kg). Diabetic group IV treated with metformin while V and VI were treated with metformin in a combination of the two doses of NPs (5 and 10 mg/kg) according to the treatment schedule. Biochemical and histological analysis of blood and liver samples were conducted after the treatment. The groups V and VI treated with the combination exhibited remarkable improvement in fasting glucose, lipid profile (HDL and cholesterol), liver function tests (AST, ALT), toxicity markers (GGT, GST and LDH), and redox markers (GSH, MDA and CAT) in comparison to group II in most of the parameters. Histological evaluation and comet assay further consolidate these biochemical results, pleading the restoration of the cellular structure of the target tissues and their nuclear DNA. Therefore, the present study shows that the NPs can enhance the anti-diabetic action by suppression of the drug-mediated hepatoxicity via relieving from oxidative stress, toxic burden and inflammation.

## 1. Introduction

Diabetes mellitus (DM) is one of the front-line global health challenges because of widespread prevalence and clinical compromises in the patients. According to IDF Diabetes Atlas Ninth Edition 2019, 463 million adults live with diabetes globally, in which almost 4.2 million people have lost their lives, making the disease the fourth leading cause of death worldwide [1]. Although much information is available on this disease, its origin chiefly includes obesity, a sedentary lifestyle, and unhealthy eating habits. However, family history, race/ethnicity, geographical location, age, other clinical conditions, and metabolic abnormalities are other factors that make people susceptible to the disease [2]. If not appropriately addressed, the disease can lead to severe clinical problems such as cardiovascular diseases, hypertension, renal failure, retinopathy, diabetic foot, neurological disorders, and infections, compromising quality of life and shortening life span [3]. The disease is attributed to a multifactorial metabolic disorder characterized by either decreased insulin secretion and/or its action or reduced uptake by the target cells, leading to hyperglycemia [4].

Metformin (MTF) is one of the most widely prescribed drugs for treating DM. It has been found to improve glucose tolerance in patients by decreasing basal and postprandial plasma glucose [5]. This drug is being used by almost 120 million type II diabetic patients worldwide, as per the latest studies [6,7]. Despite the approval of many other antidiabetic agents like thiazolidinediones, sulfonylureas, dipeptidyl peptidase-4 inhibitors, sodium-glucose cotransporter 2 inhibitors, and glucagon-like peptide-1 receptor agonists in the drug market, MTF is still the most preferred drug for DM by contemporary doctors [8]. One of the reasons behind this notion is that the drug prevents diabetes-induced cardiovascular complications [9]. The drug exerts certain major side effects including deficiency of vitamin B_12_ and folate and elevation in homocysteine concentrations that can lead to cardiovascular disease besides renal failure and liver dysfunction [7,10]. Hence, many contemporary investigators are discoursing novel methods to enhance the drug’s efficacy with fewer side effects [11].

Nanotechnology is one of the fastest-expanding fields of research in science and technology. Hence, nanoparticles (NPs) are highly applauded as one of the best ways for drug delivery with high sustenance, specificity and efficacy in the living system [5,7,12]. N.P.s as a drug delivery system offers various advantages, including a larger surface-to-mass ratio, increased drug stability, high payload capacity, extended circulation time in the blood and reduced toxicity with respect to standard capsulated or tablet versions of any medicine [13]. Silver nanoparticles (AgNPs) are one of the most promising metal-based NPs in various fields, including the drug and pharmaceutical industry [14,15,16].

The phytocompounds with pharmacological properties have been thrust areas among contemporary investigators in designing novel drugs to address diseases like diabetes and cancer [17,18]. Curcumin (Cur), diferuloylmethane, is an organic compound with active polyphenols. It is derived from the rhizomes of the plant turmeric (Curcuma longa), used as a general spice in many Asian cuisines and as a food-preserving agent. Curcumin is a biologically active compound with a rich repertoire of health benefits, including immunity and physiological improvement [19]. Many investigators have applauded it for treating chronic diseases involving inflammation, oxidative stress and internal injuries, including T2DM and cancer [18]. However, the compound’s therapeutic and pharmacologic prospective is limited because of its lower bioavailability attributed by decreased absorption in the small intestine with rapid degradation and faster systemic elimination [20,21]. Hence, since then, many novel strategies have been proposed and designed to enhance the bioavailability of curcumin in the biological system, including loading the compound as a conjugate or nanoparticle [22,23]. It is reported that curcumin in pure form, cannot be fully absorbed and utilized in vivo. Much literature suggests that curcumin, nanocarriers, and nanoparticles have shown significant anti-diabetic properties, overcoming the constraints of curcumin alone as a pharmaceutical compound [24,25,26]. Many investigators have reported that the combination of curcumin and silver nanomaterials produces a stabilized structure in the nano-size with an improved character for biological activity [26]. The present investigation aims to investigate if AgNPs-Cur (AgNPs decorated with curcumin) enhances the efficacy of metformin in streptozocin-induced DM in the rat animal model. 

## 2. Materials and Methods

### 2.1. Methods

#### 2.1.1. Animal Studies

Forty adult male rats (Wistar strain, 4–4.6 months old, 110–130 g) were used for the present investigation. Twenty eight rats were subjected to induction of diabetes mellitus by a single dose administration of streptozocin [STZ, (Abcam, Cambridge, UK)] at the dose of 50 mg/kg as previously executed [27]. The NPs were prepared as previously done [28]. The rats were categorized into the following six groups (*n* = 6):

*Group I*:  The control group without any treatment
*Group II*:  The diabetic group (a single dose of streptozocin at 50 mg/kg) without any treatment
*Group III*:  Normal rats treated with silver nanoparticles decorated with curcumin (AgNPs-Cur) at 5 mg/kg.
*Group IV*:  Diabetic rats treated with eight doses of metformin (200 mg/kg) [29].
*Group V*:  Diabetic rats treated with eight doses of metformin with AgNPs-Cur (5 mg/kg)
*Group VI*:  Diabetic rats treated with eight doses of metformin with AgNPs-Cur (10 mg/kg)

All the solutions of test chemicals were administered in the rats’ intraperitoneal (i. p.) region with an insulin syringe (1 mL capacity). The rodents were reared and taken care of under humane conditions as previously reported [30] in Animal House, Department of Zoology, KSU, Riyadh, following the guidelines of the Departmental Ethical Committee (Department of Zoology, KSU). The KSU ethical committee approved the study with approval number KSU-SE-20-38.

The rats showing fasting glucose level over 180 mg/100 mL on the third day of STZ administration were considered diabetic. Out of 28 rats dosed with STZ, 2 rats died on after 24 h, while 2 rats were found very distressed; so, they were excluded from the study

#### 2.1.2. Preparation of Silver Nanoparticles Modified with Curcumin

The chemicals applied in this research work to synthesize the silver nanoparticles-embedded curcumin, including curcumin, silver nitrate, polyvinylpyrrolidone and ethylene glycol, were purchased from Sigma-Aldrich (St. Louis, MO, USA). The procedure of [31] was employed as a guide for fabricating the silver nanomaterials. The silver nitrate was well dissolved in ethylene glycol containing polyvinylpyrrolidone stirred for 2 h. In a parallel step, the curcumin solution was prepared in ethylene glycol with a concentration of 0.1 ppm. 100 mL of the curcumin solution was rapidly added to the reaction mixture and homogenized maintaining the temperature of the reaction mixture at 100 °C for 3 h [18,19]. Finally, the formed silver nanoparticles-embedded curcumin was precipitated in acetone solution, separated by centrifugation, washed, and stored until use.

##### Characteristics of the Silver Nanoparticles-Embedded Curcumin

**Figure 1:**
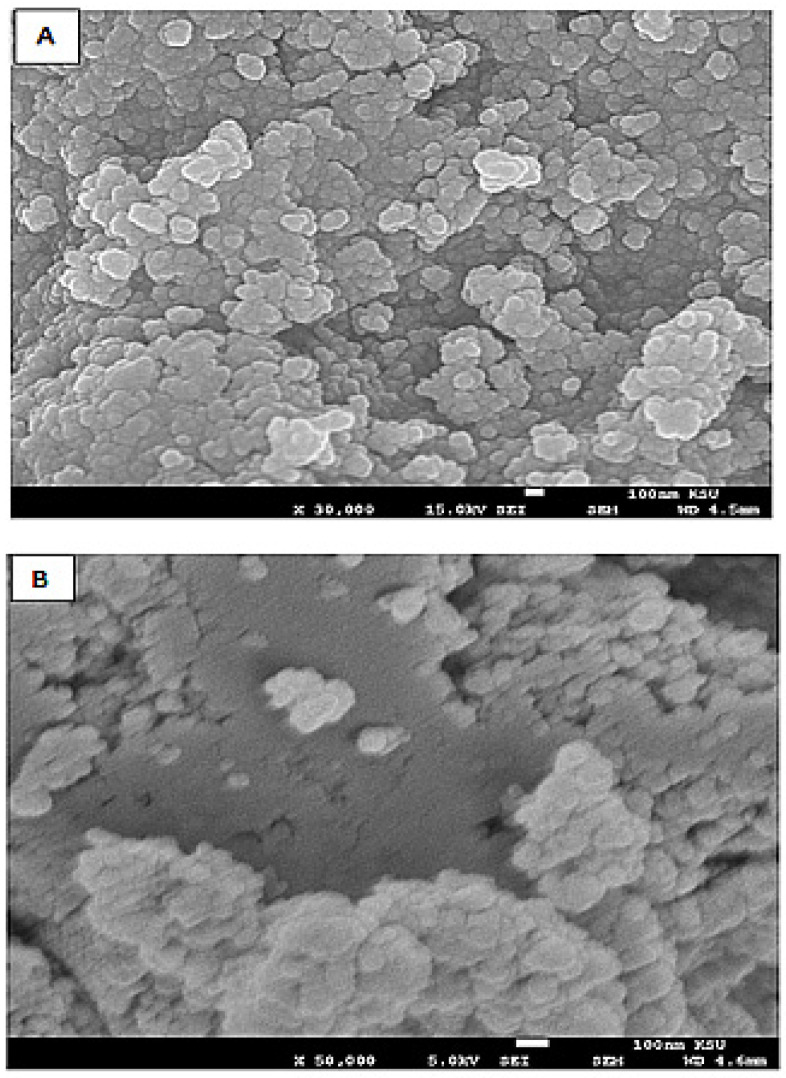
Showing SEM pictures of silver nanoparticles embedded with curcumin at the magnification 30,000× (**A**) and 50,000× (**B**).

The application of curcumin as an additive and stabilizing agent during the formation of silver nanoparticles produced a spherical shape particle with a uniform distribution, as evidenced by the SEM examination (Figure 1). These figures show that the particle size of the prepared materials is ~100 nm which vividly confirms the successful synthesis of the silver nanoparticles decorated with curcumin (AgNP-curcumin). These particles were found to be consistent in their structure and size. They were used in vivo studies to investigate its efficacy in enhancing the antidiabetic activity of metformin.

Moreover, an FTIR analysis (as a Supplementary Figure S1) of the NPs indicates the presence of C=O and OH groups on the surfaces of the hybrid materials as nanocomposite which correlates with curcumin and confirm the successful incorporation of curcumin in the formed nanoparticles.

#### 2.1.3. Preparation of Biological Samples

The blood and liver tissue samples were prepared after sacrificing, as previously reported [32,33]. The serum samples were retrieved by centrifuging at 1000× *g* while the supernatants from tissue samples were retrieved from 3000× *g*. The samples were kept at −80 °C (Eppendorff, Stevenage, UK) till further analysis [30].

#### 2.1.4. Glucose Test for the Evaluation of Diabetes

The blood samples were retrieved from the tail vein to measure the blood glucose (at 7–8 a.m.) in the food-deprived (5 p.m. to 7 a.m.) rats using a blood glucose monitoring system (Contour brand, Bayer, Tokyo, Japan). Only the rats showing fasting glucose levels over 180 mg/100 mL were categorized as diabetic. Their glucose measurement was checked twice to confirm the sustenance of the diabetic condition of the animals.

#### 2.1.5. Measurement of Glucose Level

Serum glucose level was measured by the commercial kit (QCA kits, Amposta, Spain) following the manufacturer’s instructions.

#### 2.1.6. Assessment of Lipid Profiling

Cholesterol and high-density lipids (HDL) were studied for lipid profiling by measuring their level in serum with the commercial kit (QCA kits, Spain) following the instruction manual.

#### 2.1.7. Measurement of Redox Parameters

The reduced glutathione (GSH) and malondialdehyde (MDA) were chosen as the redox parameters for evaluating their role in management of oxidative stress in vivo. GSH level was estimated by the method based on using sulfosalicylic acid and DTNB and the absorbance of the reaction mixture was read at 412 nm [34]. MDA was estimated by Beuge and Aust protocol [35] which was based on the reaction with trichloroacetic acid (TCA) and 2-thiobarbituric acid (TBA). 

#### 2.1.8. Analysis of Liver Function Markers

The serum samples were used for the assessment of liver function markers- aspartate transaminase (AST), alanine transaminase (ALT), alkaline phosphatase (ALP) and total bilirubin. The serum markers were measured by commercial kits (QCA, Spain) per the maker’s instructions. 

#### 2.1.9. Histopathological Analysis

The dissected liver tissues were kept in 10% formalin. They were cut into sections with thickness of 5–7 µM, followed by staining with H & E [14,23]. The prepared slides’ evaluation was executed blindfolded under a light microscope (Leica DMRB/E Heerbrugg, Switzerland) coupled with an HD camera (Leica MC 170 HD, Singapore). The histomicrographs of the sections were snapped at 400× and further digitally enhanced by Adobe Photoshop (Adobe Systems, Mountain View, CA, USA).

#### 2.1.10. Comet Assay

As previously done, the single-cell suspension from tissues was prepared and the assay was conducted in alkaline condition [15,32]. Fully frosted slides (Kawamoto, Japan) were layered with 1% NMA at 60 °C. 100 µL of working cell suspension (in 1% LMPA) was pipetted over the base layer and covered with cover glass (Blue star, Mumbai, India) immediately. Then, a final layer of 0.5% LMPA (80 µL) was pipetted over, covered with coverslips, and kept on ice packs again to fix the layers. The slides were submerged in chilled alkaline lysing solution (2.5 M NaCl + 100 mM EDTA + 10 mM tris-base + 1% triton X-100, pH 10) for 3 h. After that, the slides were kept in an electrophoretic buffer (300 mM NaOH + 1 mM EDTA, pH 13) in an electrophoretic tank for 30 min for unwinding. Then, electrophoresis was conducted for 35 min at 4 °C with constant field strength (0.74 volts/cm) and current strength of 300 mA. After the stoppage of the power supply, the slides were carefully washed with chilled saline and immersed in neutralizing buffer (0.4 M tris-base, pH 7.5) followed by their washing with cold saline. After three repeats of neutralization, the slides were stained with ethidium bromide (20 mg/mL) for 7 min. The slides were finally given three washes and kept in a humidified slide box (Kartell Labware, Italy). The slides were evaluated under a fluorescent microscope (DM2500, Leica, Germany) coupled with an image analysis system (Komet 5.5, Kinetic imaging, Liverpool, UK) attached to an integrated CC camera. Comet tail-length was chosen to assess nuclear DNA damage in the present study. 

### 2.2. Statistical Analysis

The data shown in the work is as mean ± S.D. analysed by GraphPad Prism 5 software, including one-way ANOVA analysis with Tukey’s post hoc multiple comparison test. The asterisk marks “*, * and *” were used to show a significant difference from negative control (C.N.) at P less than 0.05, 0.005, and 0.001. The marks “# and #” were used as asterisk marks to show a significant difference from positive control (DM) at P less than 0.05, 0.005, and 0.001. 

## 3. Results

### 3.1. Effect on Fasting Glucose Level

**Figure 2:**
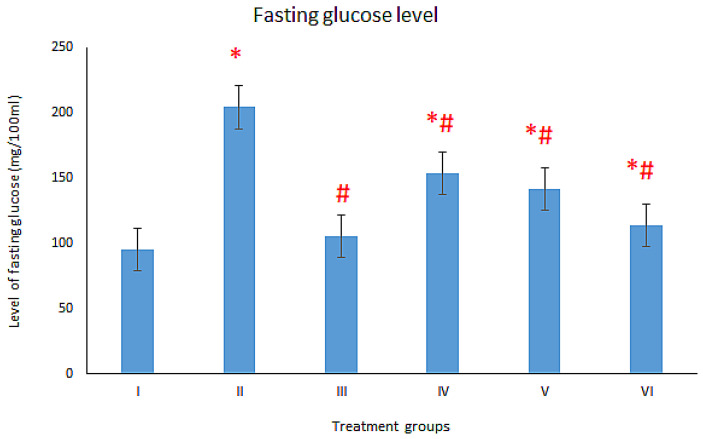
Showing bars of fasting glucose level of six different treatment groups (*n* = 6) in mg/dL estimated by the commercial kit (QCA, Spain). All the data has been shown in Mean ± SD of six different samples. The asterisk mark “*” is to show a significant difference from negative control (Group I) at P less than 0.001. The asterisk mark “#” is to show a significant difference from positive control (DM, group II) at P less than 0.001.

To assess the diabetic condition, fasting glucose level was assessed in the serum samples. Diabetes-induced group II showed an elevation in fasting glucose by 114.20% compared to the control group I. Upon treatment with MTF, group IV demonstrated a decline in its level by 24.74% with respect to group II. The treatment with MTF and silver nanoparticles (AgNP-curcumin) treatment caused a significant decrease in the glucose level as group V exhibited a decline in its level by 30.61%, while group VI displayed 44.25% decline in the level in comparison to group II (Figure 2). Hence, the NPs improved the anti-diabetic efficacy of metformin in a dose-dependent way. 

### 3.2. Effect on Liver Function Markers

The treatment exerted its effect on the standard liver function markers (ALT, AST and ALP) whose details are as follows:

#### 3.2.1. ALT

**Figure 3:**
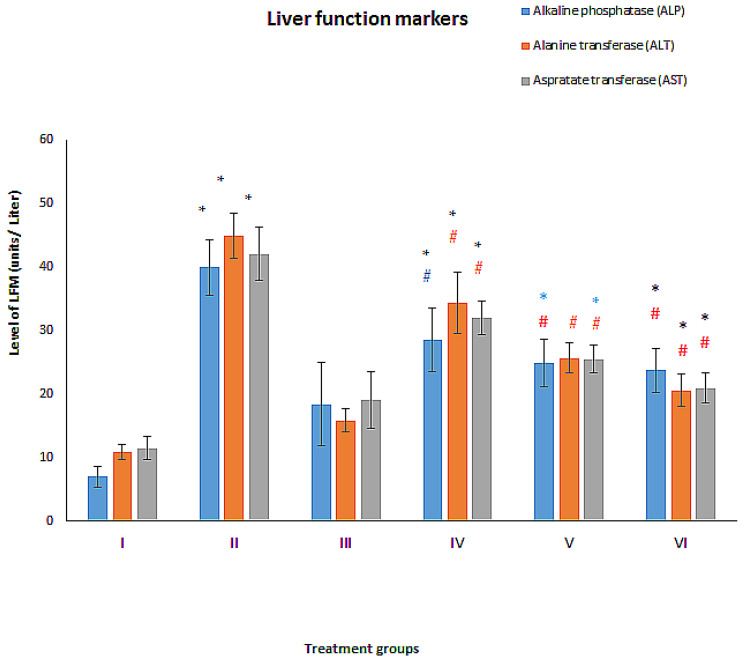
Showing bars of liver function markers of six different treatment groups (*n* = 6) in mg/dL estimated by the commercial kits. All the data has been shown in Mean ± SD of six different samples. The asterisk marks “* and *” are to show a significant difference from negative control (Group I) at P less than 0.05, 0.005, and 0.001. The asterisk marks “# and #” are to show a significant difference from positive control (DM, group II) at P less than 0.05, 0.005, and 0.001.

The diabetic group without treatment (Group II) showed a staggering elevation in its activity by 314.69% while group III (AgNPs-Cur) demonstrated an increase in its level by 46.76% compared to the control, group I. However, the combination-treated diabetic groups, group-V and VI showed a decline its activity by 42.81% and 54.08%, respectively. (Figure 3). 

#### 3.2.2. AST

The diabetic group II and the decorated NPs-treated, group III exhibited a rise in its level by 265.19% and 65.18% with respect to the control. The group IV, V and VI showed a declination in its level by 50.20%, 25.05% and 39.41%, respectively, compared to untreated diabetic group II (Figure 3). 

#### 3.2.3. ALP

The diabetic group II, and group III displayed an elevation in the level of ALP by 471.24% and 163.23% as compared to the control. In contrast, groups V and VI revealed a decrease in the level by 37.79% and 40.54% with respect to the untreated diabetic group II (Figure 3). 

### 3.3. Effect on Hepatotoxicity Markers

#### 3.3.1. Total Bilirubin

The diabetic untreated group II showed an enhanced level of this marker by 107.54% while AgNP-treated group III displayed 39.62% elevation in its level as compared to the control. However, the treatment groups-IV, V and VI declined by 2.721%, 9.09% and 20.90% compared to group II (Table 1). 

#### 3.3.2. Gamma-Glutamyl Transferase (GGT)

Group II and III displayed an elevation in its level by 470.84% and 86.38% while group IV, V and VI demonstrated a depression of 24.75%, 38.33% and 48.15% compared to group II (Table 1). 

#### 3.3.3. Glutathione–S-Transferase (GST)

Group II and III revealed an increase in its activity by 152.92% and 83.61% with respect to the control, whereas group IV, V and VI manifested a decline by 10.49%, 14.18% and 33.08% as compared to group II (Table 1).

#### 3.3.4. Lactate Dehydrogenase (LDH)

Group II and III demonstrated an enhancement in their activity by 195.88% and 58.35% compared to the control. However, group IV, V and VI showed a dip in their activity by 30.16%, 47.05% and 54.84% with respect to group II (Table 1). 

### 3.4. Effect on Oxidative Stress Markers

#### 3.4.1. Reduced Glutathione (GSH)

The diabetic untreated group II and the nanoparticles treated group III showed a decrease in its level by 92.11% and 31.92% as compared to the control, group I. Hitherto, group IV, V and VI exhibited replenishment in its level by 485.36%, 548.78% and 726.82% with respect to group II (Table 2). 

#### 3.4.2. Total Malondialdehyde (MDA)

Group II and III revealed elevated MDA levels by 212.19% and 68.29% compared to group I whereas group IV, V and VI showed dip in its level by 25%, 45.11% and 50.78% with respect to group II (Table 2).

#### 3.4.3. Catalase

The activity of this antioxidant enzyme was found depressed by 64.87% and 30.38% in group II and III when compared to the control value. In contrast, group IV, V and VI showed enhancement in its activity by 21.73%, 50.90% and 87.12% respectively (Table 2).

### 3.5. Effect on Lipid Profile

#### 3.5.1. Cholesterol

**Figure 4:**
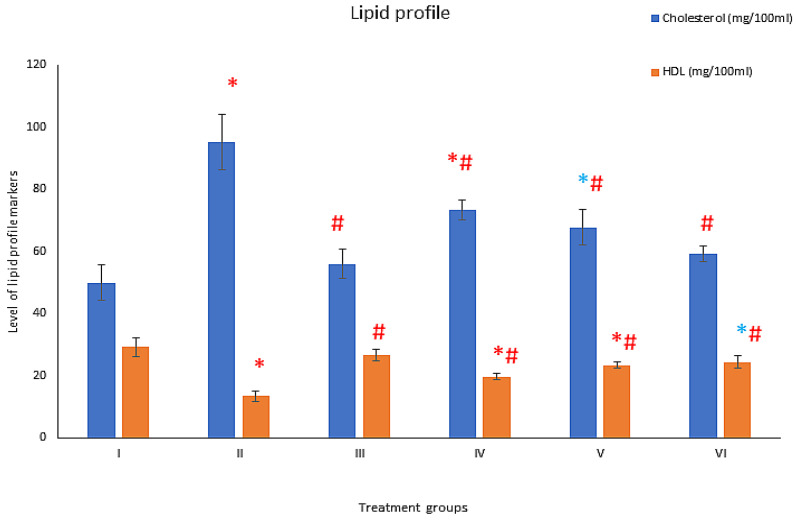
Showing bars of lipid profile markers of six different treatment groups (*n* = 6) in mg/dL estimated by kit. All the data has been shown in Mean ± SD of six different samples. The asterisk marks “* and *” are to show a significant difference from negative control (Group I) at P less than, 0.005, and 0.001. The asterisk mark “#” is to show a significant difference from positive control (DM, group II) at P less than 0.001.

The cholesterol level was elevated by 90.26% and 12.08% in groups II and III compared to group I. However, group IV, V and VI exhibited a decline in its level by 22.94%, 28.52% and 37.67%, respectively concerning group II (Figure 4).

#### 3.5.2. High-Density Lipids (HDL)

The HDL level was devitalized in groups II and III by 54.18% and 8.97% compared to group I. In contrast, group IV, V and VI exhibited an increase in level by 47.42%, 74.24% and 81.89%, respectively, compared to group II (Figure 4).

### 3.6. Histological Evaluation

**Figure 5:**
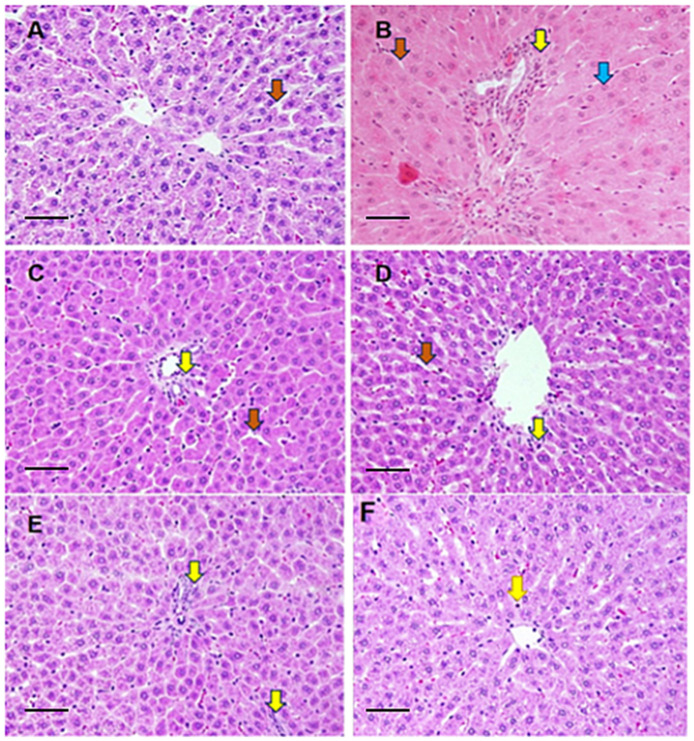
Representative photomicrographs of the hepatic tissues from control (**A**), diabetic from group II (**B**), diabetic treated with metformin from group III (**C**), AgNPs-Cur treated group IV (**D**), diabetic treated with metformin with the NPs at 5 mg/kg and 10 mg/kg in group V and VI (**E**,**F**). The major histological alteration indicated the hepatocytes (blue arrows), sinusoidal space (brown arrows) and inflammatory cells (yellow arrows). The sections were snapped under a light microscope (Leica, St. Gallen, Switzerland) at the magnification 400× with an HD camera (Leica MC 170 HD, Singapore) at a scale bar of 100 µm.

The hepatic architecture of various treated groups has been shown in Figure 5. Figure 5A depicts the liver’s control micrograph (group I) with the normal distribution of polygonal hepatocytes with typical nuclei. The blood sinusoids have typically appeared with peripheral Kupffer cells. On the opposite side, severe pathological alterations were remarked in the hepatic tissues of the diabetic rats (Figure 5A). Histological evaluation of this group showed that the hepatocytes have eosinophilic cytoplasm without any marked granules compared to the normal sections. Due to the abnormal distribution of the diabetic hepatocytes, the sinusoidal spaces appeared narrow than normal. In addition, the hepatic tissues in diabetic rats are extensively infiltrated with inflammatory immune cells (Figure 5B). An improvement is observed in the hepatic tissues of the diabetic rats after the treatment with the antidiabetic drug metformin. This group’s examination showed moderate histopathological changes such as mild infiltration of the inflammatory cell and the narrow sinusoids (Figure 5C). It is evident from Figure 5D that shows a histomicrograph with minimal histological changes in the hepatic tissues from the rats treated with silver nanoparticles with curcumin (AgNP-Cur) and was quite comparable to the control group (5A). Wide sinusoids with slight inflammatory cell infiltration are observed in the sliver nanoparticles’ hepatic tissues with curcumin rats (Figure 5D).

On the other hand, the diabetic rats treated with metformin and AgNP-Cur (Figure 5E,F) showed remarkable improvement in the two doses of the NPs (5 and 10 mg/kg). However, minimal changes were still observed in these rats’ tissues, such as the inflammatory cell infiltration. Figure 5E,F show dose-dependent histological restoration. Figure 5F seems quite comparable to the control despite mild histological distortion.

### 3.7. Comet Assay

**Figure 6:**
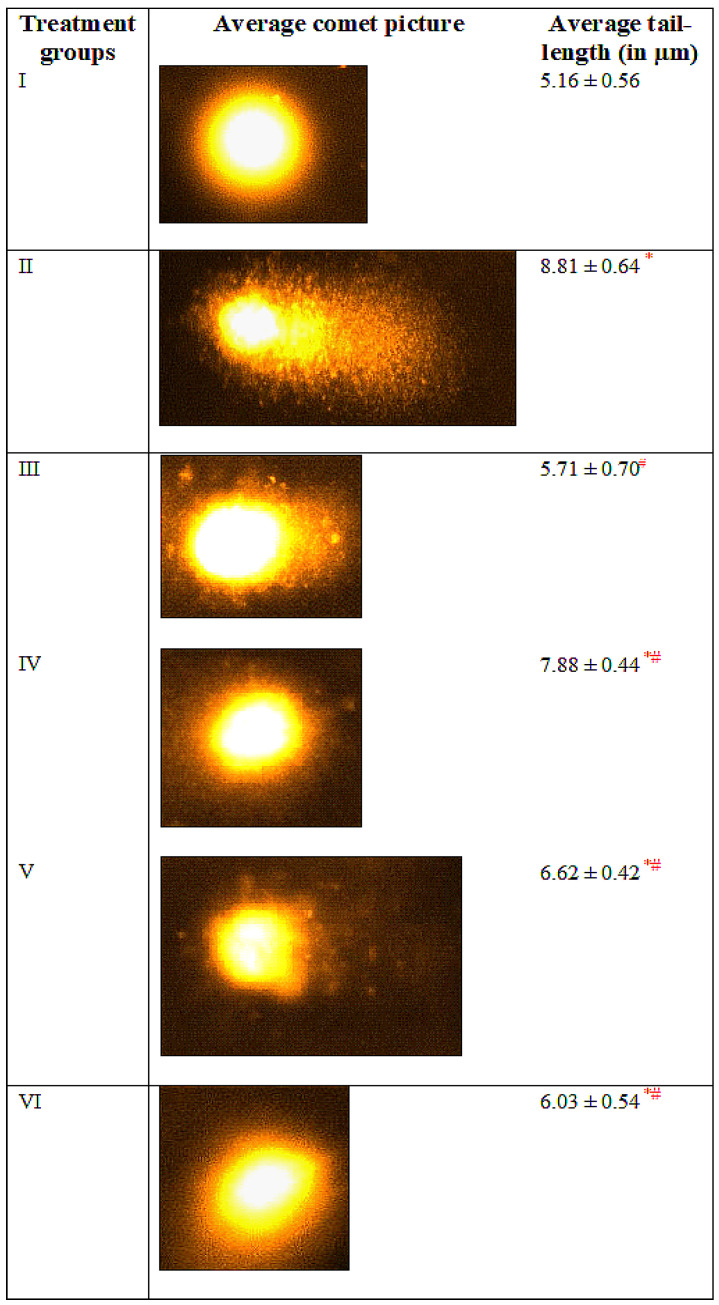
The figure shows the average comet picture of different groups along comet tail-lengths (in micrometer) by Komet 5.5 imaging software (Oxford, UK). The asterisk mark “*” is to show a significant difference from negative control (Group I) at P less than 0.001. The asterisk mark “#” are to show a significant difference from positive control (DM, group II) at P less than 0.001.

The diabetic untreated group II showed an increase in tail length by 70.73% with respect to the control while the nanoparticles alone treated, group III demonstrated merely 10.65% of increment. Notably, the NPs did not exert any derogatory effect on nuclear DNA of the target cells. The combination-treated groups-V and VI exhibited a decrease in the tail-length by 24.85% and 31.55% as compared to group II (Figure 6). 

## 4. Discussion

DM is one of the major diseases that claim most human lives after cardiovascular diseases and cancer, as per mortality data issued by CDC, United States in 2019. Many investigators have suggested numerous ways to manage and control this lifestyle-related disease [2,7,21,36]. However, despite advancing science and technology progress, the disease cannot be tamed. The present investigation proposes a combined treatment strategy against the disease by enhancing the pharmaceutical potency of the most established antidiabetic drug, metformin, with the application of nanotechnology. The study results are prudent in showing significant enhancement of the antidiabetic potency of metformin by AgNP-curcumin in DM-induced rats. The diabetic rats showed substantial hyperglycemia, elevated liver function, and toxicity markers concomitant with altered lipid profile and oxidative stress markers. The diabetic rats treated with MTF alone showed improvement in many parameters; however, the combinations of MTF with AgNP-Curcumin demonstrated significant improvement in most of the parameters in a dose-dependent manner. Further, the comet assay and histological evaluation were consistent with the biochemical measurement, consolidating these results.

It is established that streptozocin (STZ) infiltrates the pancreas and invades insulin synthesizing β-cells that subsequently leads to diabetes mellitus (DM) induction evidenced by hypoinsulinemia and hyperglycemia within a week after its administration in the experimental animal models [29,37]. Most investigators elucidate that the reactive oxygen species (ROS) triggered by STZ are the chief causative agents in STZ-induced DM’s pathogenesis in vivo [29,38]. Additionally, the high glucose level gets auto-oxidized in diabetic conditions, further worsening the redox balance in vivo [39]. After induction of DM, the ROS trigger the cascade of hepatotoxicity mediated by heightened oxidative stress and inflammatory response. Also, the ROS react with the lipid, causing enhanced lipid peroxidation as evidenced by elevated MDA levels in the present investigation. Hence, the ROS, being aggressive tiny radicals, can penetrate through the cell membrane and all the membraned organelles (mitochondria, E. R., Golgi bodies, nucleus) in the target tissues [15]. Herein, DM-induced ROS also invades the liver’s hepatocytes, leading to extensive tissue damage that causes leakage of various biochemical markers into the blood [40]. In the current investigation, the diabetic untreated rats demonstrated an elevation liver function test markers (AST, ALT and ALP) in the serum samples. Further, after accumulating this liver damage, the toxicity markers (LDH, ALP, GST and GGT) also rise in the serum samples [41] as evidenced in the present study. In addition, these radicals exhaust the reservoir of antioxidant enzymes and proteins (CAT and GSH) in chronic diabetic conditions. Finally, ROS make their way to the nucleus and affects the nuclear DNA, proteins and carbohydrate in the milieu of target tissues. It is obvious from the increased tail-length of nuclear DNA and distorted histological details in the diabetic untreated group. After treatment with the first line of the antidiabetic drug, metformin, the studied parameters were somewhat improved in diabetic rats. Intriguingly, the co-administration of AgNP-curcumin with metformin substantially improved the diabetic condition in a dose-dependent manner against the metformin alone treated rats to a significant level.

Recently, Saratale et al. [42] have reported curcumin along with metformin act synergistically in countering oxidative stress and dyslipidemia in diabetic rats. Our study also demonstrates similar effects, although the inclusion of AgNPs loaded with curcumin exerted profound improvement in most of the diabetic rats. It is documented that curcumin has a wide horizons of selected pharmacological and medicinal properties including antidiabetic, antioxidant, anti-inflammatory, anti-allergies and anti-lipedema, besides anticancer activity [21,43]. These suitable features make curcumin a great candidate for diabetes treatment. Also, it is reported that a large group of pre-diabetic individuals on set to become type 2 diabetic showed significant improvement in the pancreatic beta-cell function and related glycemic indexes after treatment with curcumin for nine months [17]. Hitherto, a similar report showing improved fasting, postprandial glucose and insulin sensitivity, this natural product’s pharmacological application is limited because of various factors, including poor solubility, low bioavailability, and compromised efficacy after interaction with other biomolecules in vivo before reaching the target cells [44]. Hence, novel formulations or methods to enhance its effectiveness have been highly desired among investigators for long [21,43]. In the present study, silver nanoparticles (AgNPs) have been employed to counter curcumin’s mentioned limitations in vivo. Many studies have revealed that AgNPs possess superior anti-glycemic and insulin sensitivity-enhancing activities [45,46]. Therefore, the administration of metformin with AgNP-curcumin exhibits tremendous antidiabetic potential in the present investigation. The combination also suppresses oxidative stress and lowers lipid peroxidation, restoring cellular structures and functions. Consequently, it can also restore the histology of the pancreatic islets and their functions, which might replenish the activity level of insulin and its sensitivity [44]. It is also documented that the antioxidant feature of both adjuvants (curcumin and NPs) can cease the inflammatory aggression of the free radicals, evidenced by the lowering of pro-inflammatory interleukins (IL-1, 6) and modulation of PON1 activity [42]. Also, curcumin has been reported to downgrade inflammation and trigger apoptosis in the damaged pancreatic beta cells, resulting in improved function and glucose homeostasis [47]. The body might learn to manage and cope with raised glucose levels after appropriately orchestrating redox balance and physiology by the proposed combination. The comet assay further confirms the restoration of nuclear DNA with the administration of the combination. 

Metformin is a primary advanced anti-diabetic drug with multiple complicated molecular mechanisms and numerous drug targets inside the target cells. The drug exerts its physiological effect on the liver directly or indirectly, leading to declination in gluconeogenesis with enhancing effect on glucose uptake by the gut concomitant with an increase in glycogen synthesis along with alteration of the gut normal flora. On the other hand, the drug at the molecular level hinders the mitochondrial respiratory chain (by inhibiting complex 1) in the liver. This triggers the activation of AMPK, increasing insulin sensitivity and lowering cAMP which consequently downregulates the enzymes of gluconeogenesis. In addition, metformin can act on the liver in an AMPK-independent way, including inhibition of fructose-1,6-bisphosphatase by AMP [48]. It is well known that AgNPs-curcumin, like other NPs after getting into the biological system, there is leaching-out of curcumin along with the release of Ag as ions (Ag^+1^) from the NPs. Wahab et al. [49] have shown that the NPs can restore the diabetes-induced histological alteration in the target organs (liver, pancreas and kidney). Also, curcumin has well-proved antioxidant and anti-inflammatory properties that facilitate the healing of β-cells of the pancreas and overall metabolism of the diabetic condition [18]. It is documented that the bioactive compound can promote programmed cell death by inhibition of intracellular transcription factors including NF-κB, activator protein 1, cyclooxygenase II (COX-2), nitric oxide synthase, MMP-9, and STAT3 [50]. It is established that redox imbalance and ROS-mediated inflammation contribute to the etiology of DM. Hence, it is speculative that the combination of AgNPs with curcumin will not only reverse the key events leading to diabetic conditions but also aid in the efficacy of metformin. In the present investigation, the AgNPs-Curcumin orchestrate the redox status of the target organs (liver and pancreas) by enhancing the activity of antioxidant enzymes and proteins that decrease the toxic burden and inflammation in liver and pancreas [50]. It improves the functionality of the pancreas (insulin synthesis and section) and the liver (enhancement of glucose uptake and glycogen synthesis). In addition, AgNPs have been reported to enhance insulin sensitization via the rise in cytosolic Ca^++^ and activation of AMPK by phosphorylation in vitro as well as in vivo, consequently increasing insulin sensitivity and its action [51]. This cascade also improves glucose uptake by the target cells by reinforcing the insulin receptors (IRS1) and GLUT2 expression. Hence, the NPs can enhance the efficacy of metformin via multiple molecular mechanisms. 

## 5. Conclusions

Metformin with AgNPs-curcumin downregulates diabetes-induced oxidative stress and altered immune response-mediated hepatotoxicity towards the normal in vivo. It starts the cascade of reversal of diabetic indexes, including fasting glucose, lipid profile, and restoration of hepatic tissues improving the functionality of liver and the pancreas consequently enhancing insulin availability, sensitivity and response at the target tissues. However, further studies are warranted to investigate the in-depth mechanism involved at molecular and genetic level.

## Figures and Tables

**Table 1:** Showing hepatotoxicity markers of six different treatment groups (*n* = 6) in units in parentheses. All the data has been shown in Mean ± SD of six different samples. The asterisk marks “* and *” are to show a significant difference from negative control (Group I) at P less than 0.05, 0.005, and 0.001. The asterisk marks “#” are to show a significant difference from positive control (DM, group II) at P less than 0.05, 0.005, and 0.001.

Treatment Groups	Gamma Glutamyl Transferase (U/L)	Glutathione-S-Transferase (Units/mg)	Lactate Dehydrogenase (U/L)	Total Bilirubin (µmol/L)
Control (Group I)	78.51 ± 12.41	16.42 ±3.32	71.04 ± 16.94	5.32 ± 0.76
Diabetic group (Group II)	448.10 ± 64.17 *	41.53 ± 6.66 *	210.21 ± 24.22 *	11.06 ± 0.59 *
AgNPs-Curcumin group (Group III)	146.30 ± 27.61 #	30.15 ± 2.35 *	87.53 ± 10.07 #	7.47 ± 0.90 *
Diabetic group + MTF (Group IV)	373.17 ± 56.41 *	37.17 ±3.96 *	146.80± 7.62 *#	10.70 ± 0.76 *#
Diabetic group + MTF+ AgNP-Curcumin (5 mg/kg) (Group V)	276.30 ±65.23 *#	35.64 ± 2.28 *	111.30± 1.36 *#	10.01 ± 0.24 *
Diabetic group + MTF+ AgNP-Curcumin (10 mg/kg) (Group VI)	233.0 ± 35.94 *#	27.79 ± 2.13 *#	94.91 ± 6.05 #	8.78 ± 0.59 *#

**Table 2:** The table Showing level of oxidative stress markers of six different treatment groups (*n* = 6) in mg/dL estimated by kit. All the data has been shown in Mean ± SD of six different samples. The asterisk marks “*, * and *” are to show a significant difference from negative control (Group I) at P less than 0.05, 0.005, and 0.001. The asterisk marks “# and #” are to show a significant difference from positive control (DM, group II) at P less than 0.05, 0.005, and 0.001.

Treatment Groups	MDA (nmol/mg)	GSH (nmol/mg)	CAT (units/mg)
Control (Group I)	0.164 ± 0.040	0.528 ± 0.077	5.662 ± 0.868
Diabetic group (Group II)	0.512 ± 0.053 *	0.0413 ± 0.003	1.988 ± 0.246 *
AgNPs-Curcumin group (Group III)	0.276 ± 0.016	0.354 ± 0.068 *#	3.943 ± 0.439 *
Diabetic group + MTF (Group IV)	0.384 ± 0.059 *#	0.240 ± 0.035 *	2.423 ± 0.332 *#
Diabetic group + MTF + AgNP-Curcumin (5 mg/kg) (Group V)	0.281 ± 0.0192 *#	0.266 ± 0.029 * #	3.002 ± 0.347 *#
Diabetic group + MTF + AgNP-Curcumin (10 mg/kg) (Group VI)	0.252 ± 0.038 *#	0.339 ± 0.007 *#	3.721 ± 0.150 *#

## Data Availability

All the relevant data are included in the manuscript.

## Supplementary Materials

The following supporting information can be downloaded at: https://www.mdpi.com/article/10.3390/toxics11100867/s1, Figure S1: Showing FTIR of the sliver nanoparticles decorated with curcumin.
