# Supplementary figures and images for: Silver Nanoparticles Decorated with Curcumin Enhance the Efficacy of Metformin in Diabetic Rats via Suppression of Hepatotoxicity
Source: Toxics. 2023 Oct 18;11(10):867. doi: 10.3390/toxics11100867 (PMC10611133; doi:10.3390/toxics11100867)

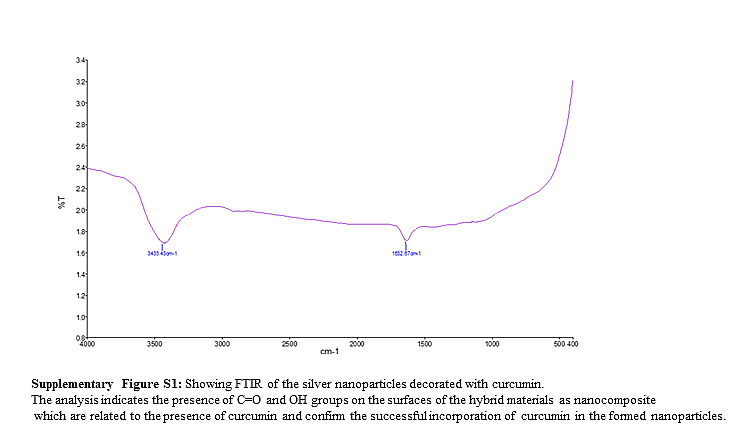

Supplement: Supplementary file 1 [file toxics-11-00867-s001.zip › toxics-2614418-supplementary.tif]
